# Terahertz Frequency-Scaled Differential Imaging for Sub-6 GHz Vehicular Antenna Signature Analysis

**DOI:** 10.3390/s20195636

**Published:** 2020-10-02

**Authors:** Jose Antonio Solano-Perez, María-Teresa Martínez-Inglés, Jose-Maria Molina-Garcia-Pardo, Jordi Romeu, Lluis Jofre-Roca, Christian Ballesteros-Sánchez, José-Víctor Rodríguez, Antonio Mateo-Aroca

**Affiliations:** 1 Departamento Tecnologías de la Información y las Comunicaciones, Universidad Politécnica de Cartagena, Cartagena, 30202 Murcia, Spain; josemaria.molina@upct.es (J.-M.M.-G.-P.); jvictor.rodriguez@upct.es (J.-V.R.); 2 Centro Universitario de la Defensa, Universidad Politécnica de Cartagena, Base Aérea de San Javier. Academia General del Aire, 30720 Murcia, Spain; mteresa.martinez@cud.upct.es; 3 CommSenslab, Department of Signal Theory and Communications, School of Telecommunications Engineering Technical University of Catalonia (Universitat Politecnica de Catalunya, UPC) Campus Nord UPC, Edif. D-3 Jordi Girona, 1-3, 08034 Barcelona, Spain; romeu@tsc.upc.edu (J.R.); jofre@tsc.upc.edu (L.J.-R.); christian.ballesteros@tsc.upc.edu (C.B.-S.); 4 Departamento Automática, Ingeniería Eléctrica y Tecnología Electrónica, Universidad Politécnica de Cartagena, Cartagena, 30202 Murcia, Spain; Antonio.Mateo@upct.es

**Keywords:** frequency-dimension scale, terahertz, measurements, differential imaging

## Abstract

The next generation of connected and autonomous vehicles will be equipped with high numbers of antennas operating in a wide frequency range for communications and environment sensing. The study of 3D spatial angular responses and the radiation patterns modified by vehicular structure will allow for better integration of the associated communication and sensing antennas. The use of near-field monostatic focusing, applied with frequency-dimension scale translation and differential imaging, offers a novel imaging application. The objective of this paper is to theoretically and experimentally study the method of obtaining currents produced by an antenna radiating on top of a vehicular platform using differential imaging. The experimental part of the study focuses on measuring a scaled target using an imaging system operating in a terahertz band—from 220 to 330 GHz—that matches a 5G frequency band according to frequency-dimension scale translation. The results show that the induced currents are properly estimated using this methodology, and that the influence of the bandwidth is assessed.

## 1. Introduction

The future generation of connected vehicle, along with the autonomous vehicle it evolves into, will require significantly increasing the number of antennas on its surface operating at different frequency bands from sub-6 GHz to millimeter-wave (mmWave), as effective communication and environment sensing are ensured in this way in respect of other vehicles and different base-stations [1].

The operation of these antennas and a focus on a 3D spatial angular response (3D radiation pattern) may be significantly influenced (perturbed) by the nearby vehicular structure. The task of studying these effects may require ascertaining the distribution of the currents induced by the antenna on the vehicular surface by means of numerical or experimental methods. The process of obtaining these currents for realistic vehicle geometries, especially when using experimental techniques, may require complex and bulky setups.

The new imaging systems operating in terahertz frequencies provide a very interesting tool that is applicable for solving the stated problem in relation to the estimation of the induced currents on the vehicular structure by the antenna. This imaging system provides the reconstructed image and shape obtained via the scattered field of metallic or non-metallic targets [2].

The concept of using terahertz frequencies and imaging techniques has been reviewed in order to confirm its applicability to the present problem. In [3], the ultrawide-band (UWB) imaging radar system used in this paper is tested at mmWave and terahertz bands, validating the spatial resolution on the order of millimeters and its imaging capabilities. The work presented in [4] is an imaging system that inspects a polymer radome in the frequency range of 70–170 GHz, reporting good performance in resolution and proposing to increase the frequency up to 300 GHz for improved resolution. Continuing with frequencies around 300 GHz, the three-dimensional (3D) imaging system presented in [5] is a synthetic aperture radar (SAR) operating at 340 GHz, utilizing the Fourier transform in two dimensions for the reconstruction process. In [6], the operating frequency is increased to 522 GHz. Finally, to conclude the background review, a novel reconstruction process in time domain operating in the microwave and mmWave frequency bands is presented in [7].

Extensive discussion has focused on the structural scattering and the scattering of an antenna’s radiation mode [8]. It is intended to reconstruct the currents associated with scattering of the radiation mode of an antenna.

The main objective of this paper is to investigate the distribution of currents induced by the antenna on the vehicular surface by means of differential image reconstruction processes, and using a previously developed multi-frequency system [3] operating in the terahertz frequency band and extending this concept to explore an area. This experimental method is a non-invasive way of obtaining the induced currents without placing the antenna in the supporting structure. A frequency-dimension scale translation is applied to reduce complexity from an experimental point of view. As the defining aspect, a differential imaging concept is exploited to process the images captured during the experiment. In this specific case of measurements, it could be applied to the study and optimization of the co-location of several antenna systems in vehicles. To the authors’ best knowledge, the uniqueness of this paper concerns dealing with a differential imaging method to obtain the current distribution without influencing the measure.

As discussed previously, the terahertz imaging radar system makes it possible to obtain the spatial distribution of the reflectivity of an object for the analytical and experimental assessment of induced currents in the vehicle structure. This system uses a double focal system with an independent transmitter antenna and receiver antenna, being connected to both ports of a vector network analyzer (VNA) to obtain the scattering parameters over a defined frequency range [3]. Using frequency-dimension scale translation, and as a proof-of-concept, the behavior of a car with a length of 4.5 m operating at the new 6 GHz (3.5 GHz) 5G frequency band was studied through a 1:43 scale metallic car prototype of 10.5 cm length, measured at the R-band (220 GHz–330 GHz). The 1:43 scale metallic car corresponds with a frequency range from 2.5 to 3.8 GHz. The results are differential measurements calculated from the difference between the scattering image reconstructed by the imaging system with the antenna (ON state or short-circuited monopole antenna) and without the antenna (OFF state or open-circuited monopole antenna). Then, an analysis of the differential image provides key information about the current car surface distribution.

This paper is structured as follows: Section 2 jointly develops the theoretical formulation—related to the total image and differential image reconstruction process with the measurement description. Section 3 includes the total image reconstruction for the different measurement cases. Section 4 shows the results related to differential imaging. Finally, Section 5 presents the conclusions.

## 2. The Imaging Process

In this section, the two-step process of imaging is formulated, first in terms of total image, and then in a novel differential form, to apply to the reconstruction of the currents created by a radiating antenna on top of a vehicle.

### 2.1. Formulation of the Total Image Reconstruction Process

**Figure 1:**
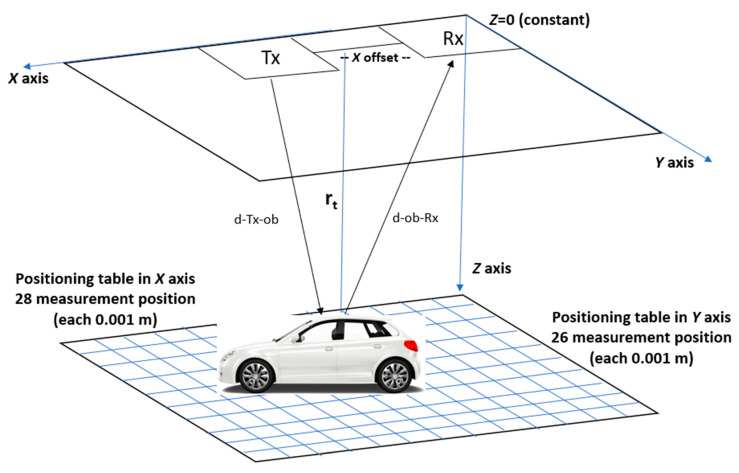
Measurement arrangements to explore the object in an *XY* plane using a positioning table.

The ultrawide-band (UWB) near-field imaging radar system used for the measurement is a flat SAR system composed of a transmitter antenna (*Tx*) and a receiver antenna (*Rx*) with fixed spatial separation, named as *X* offset distance, being a monostatic configuration but with a bi-static angle. The *Tx*–*Rx* system is fixed and explores the environment by moving the object in an *XY* plane using a positioning table. The system points toward the target located at a distance *r_t_* in the *Z*-axis, as shown in Figure 1.

Figure 1 shows the measurement arrangements, depicting the *Tx*–*Rx* and the object.

The objective is to get the spatial distribution image of the electric contrast associated with the object under test. The electric contrast is written as $c\left(\vec{r}\right)=\frac{\left(\varepsilon_{t}\left(\vec{r}\right)-\varepsilon_{b}\right)}{\varepsilon_{b}}$, with $\varepsilon_{t}\left(\vec{r}\right)$ being the object’s complex dielectric permittivity relative to its environment (background) constant value $\varepsilon_{b}\left(\vec{r}\right)$.

The imaging system generates an illuminating field with polarization in the *y*-axis (transversal) that is incident on the object under investigation, represented by a metallic car in Figure 1. $\vec{E}\left(\vec{r},f;{\vec{r}}_{t_{i}}\right)$ denotes the electric field generated in point ${\vec{r}}_{t_{i}}$ by the *Tx* antenna placed at ${\vec{r}}_{t}$ and operating at frequency f.

(1) $${\vec{J}}_{eq}\left(\vec{r},f;{\vec{r}}_{t_{i}}\right)=j\omega \varepsilon_{b}\;c\left(\vec{r}\right)\vec{E}\left(\vec{r},f;{\vec{r}}_{t_{i}}\right)$$

The equivalent current ${\vec{J}}_{eq}$ is the scattering field source generated by the object. Then, ${\vec{J}}_{eq}$ is understood as a “trace” of the original object (image).

Figure 1 shows the *Tx* and *Rx* antennas fixed in position, and the object is moved using *XY* plane geometry located at a distance of ${\vec{r}}_{t}$, on a set of positions $\left(N_{x},N_{y}\right)$ across x-axis and y-axis, to gather a 2D array that includes monostatic measurements.

For targets with highly conductive behavior ($\sigma \gg \omega \varepsilon_{0}\varepsilon_{r}^{\prime }$), like the metallic car used in this paper, the equivalent currents tend to produce the conduction current ${\vec{J}}_{eq}=\sigma \vec{E}$ [9].

The equivalent current ${\vec{J}}_{eq}\left({\vec{r}}_{t},f;{\vec{r}}_{t_{i}}\right)$ generates a scattered field (${\vec{E}}_{s}$) in the point ${\vec{r}}_{t_{i}}$ of the object, which is measured by the *Rx* antenna at each point of measurement. ${\vec{E}}_{s}$ has the following equation:

(2) $${\vec{E}}_{s}\left({\vec{r}}_{R_{j}},f;{\vec{r}}_{t_{i}}\right)=-j\omega \mu_{0}\int_{V_{0}}^{}{\vec{J}}_{eq}\left({\vec{r}}_{t},f;{\vec{r}}_{t_{i}}\right)G\left(\left|{\vec{r}}_{R_{i}}-{\vec{r}}_{t}\right|,\;f\right)dV$$

where ω is the angular frequency, $\mu_{0}$ is the magnetic permeability in vacuum, ${\vec{J}}_{eq}$ is the equivalent current, and $G\left(\left|{\vec{r}}_{R_{i}}-{\vec{r}}_{t}\right|,\;f\right)$ is the Green’s function that matches the geometry of the problem. For a 3D measurement geometry, Green’s function is written as G(r)=e−jkbrr, where $k_{b}=\omega \sqrt{\mu_{0}\varepsilon_{0}\varepsilon_{b}}$.

For the case where the object is uniformly illuminated using high-directivity antennas, ${\vec{E}}_{s}$ is written as:

(3) $${\vec{E}}_{s}\left({\vec{r}}_{R_{j}},f;{\vec{r}}_{t_{i}}\right)=-k_{b}^{2}\left(f\right)A_{d}\int_{V_{0}}^{}\;c\left(\vec{r},f\right)G\left(\left|{\vec{r}}_{t}-{\vec{r}}_{t_{i}}\right|,f\right)\;G\left(\left|{\vec{r}}_{R_{i}}-{\vec{r}}_{t}\right|,\;f\right)dV$$

where the parameters of the transmitter and receiver antennas are included in the complex constant named $A_{d}$.

The total image reconstruction process introduced by the previous works [3,9,10,11,12,13] is applied, characterized as a bi-focusing technique using multi-frequency. The contrast factor $c\left(\vec{r}\right)$ is averaged through the complete terahertz band and to the entire reconstruction space, which can be expressed as

(4) $$c\left(\vec{r}\right)=A_{o}\sum_{f_{min}}^{f_{max}}\sum_{i=1}^{N_{T-R}}\frac{{\vec{E}}_{s}\left({\vec{r}}_{R_{j}},f;{\vec{r}}_{t_{i}}\right)}{k_{b}^{2}\left(f\right)}e^{jk_{b}^{}\left(f\right)\left(\left|{\vec{r}}_{t}-{\vec{r}}_{t_{i}}\right|+\left|{\vec{r}}_{R_{i}}-{\vec{r}}_{t}\right|\right)}$$

where the parameters of the transmitter and receiver antennas are included in the complex constant called $A_{o}$. In order to reduce the computation times, fast Fourier transform (FFT) is used to solve Equation (4).

### 2.2. Formulation of Differential Image Reconstruction Process

The two-step process, described in the previous paragraph, is composed of a first step—the direct problem of obtaining the scattering fields produced by the external illumination responsible for the creation of the currents into the object—and a second step—an inverse problem focusing back on the previously obtained scattered fields to obtain an image of the currents created by the nearby illuminating geometry.

Here, the goal is to obtain the currents created not by the external illuminating geometry but by the vehicle antenna close to the object, but still using the same external illuminating geometry. These vehicular antenna currents, as described below, should be considered as a differential contribution for two different states: an ON state, or short-circuited monopole antenna, installing monopole in contact with vehicle surface, and an OFF state, or open-circuited monopole antenna, removing the monopole antenna.

To extract the differential impact of in-vehicle antenna radiation, the scattering fields will be collected and the corresponding image of the currents on the surface of the vehicle will be obtained for two successive states of the vehicle antenna: short-circuited and open-circuited [14].

In the first case (the vehicle antenna in the ON, short-circuited state), the total scattered fields ${\vec{E}}_{ant-on\;}^{total}$ are produced by the combination of the scattered fields due to the currents created on the surface of the object by the external illuminating geometry ${\vec{E}}_{ant-on\;}^{ext-illun}$, the scattered fields due to the structural currents flowing into the vehicle antenna structure without interacting with its port ${\vec{E}}_{ant-on\;}^{ant-str}$, and the scattered fields due to the antenna’s radiating mode, ${\vec{E}}_{ant-on\;}^{ant-rad}$ [15]:

(5) $${\vec{E}}_{ant-on\;}^{total}={\vec{E}}_{ant-on\;}^{ext-illun}+\;{\vec{E}}_{ant-on\;}^{ant-str}+\;{\vec{E}}_{ant-on\;}^{ant-rad}$$

In the second case (vehicle antenna in the OFF, open-circuited state), the total scattered fields ${\vec{E}}_{ant-off\;}^{total}$ are produced by the combination of the scattered fields due to the currents created on the surface of the object by the external illuminating geometry ${\vec{E}}_{ant-off\;}^{ext-illun}$ and the scattered fields due to the structural currents flowing into the vehicle antenna structure without interacting with its port ${\vec{E}}_{ant-off}^{ant-str}$.

(6) $${\vec{E}}_{ant-off\;}^{total}={\vec{E}}_{ant-off\;}^{ext-illun}+\;{\vec{E}}_{ant-off\;}^{ant-str}$$

For the compact vehicle antenna, resonant antennas are utilized in most vehicles. Then, it may be considered: (i) that interactions of a high order, such as reflections among external illuminating geometry and vehicle antenna or vehicular platform, may be neglected since they are much smaller than the rest; and (ii) that the scattered fields produced by the unchanged parts, like the vehicular platform and the structure of the antenna, are approximately the same for both states of the vehicle antenna: ${\vec{E}}_{ant-on\;}^{ext-illun}≅{\vec{E}}_{ant-off}^{ext-illun}$ and ${\vec{E}}_{ant-on\;}^{ant-str}≅{\vec{E}}_{ant-off}^{ant-str}$.

Based on the previous assumption, the reconstructed currents above the vehicle surface reconstructed from the differential scattered fields ${\vec{E}}_{ant\;}^{dif}$ can be established as:

(7) $${\vec{E}}_{ant\;}^{dif}={\vec{E}}_{ant-on\;}^{total}-\;{\vec{E}}_{ant-off\;}^{total}≅\;{\vec{E}}_{ant-on\;}^{ant-rad}$$

being related to the currents produced by the radiating mode of the vehicle antenna, and Equation (4) becomes:

(8) $$c_{ant}^{dif}\left(\vec{r}\right)=f\left({\vec{E}}_{ant\;}^{dif}\right)≅\;c_{ant-on}^{total}\left(\vec{r}\right)-\;c_{ant-off}^{total}\left(\vec{r}\right)$$

### 2.3. Measurement Description and Configuration

The measurement device was a vector network analyzer (VNA) (Rohde Schwarz model ZVA 67). One VNA port was connected to the frequency converter and the *Tx* antenna in which the converter was mounted. In the same way, the other VNA port was connected to the frequency converter and the *Rx* antenna was mounted in this converter as well. The frequency converters allow us to extend the VNA operating frequency band up to 220–330 GHz [16]. The setup configuration developed in [3] measured the S_21_ parameter (scattering parameter) in the terahertz band to reconstruct the image using a UWB near-field multi-frequency bi-focusing algorithm.

The geometry for the measurement depicted in Figure 1 is used to estimate the distribution of the induced currents on a metal car of 10 cm length—using a terahertz band for imaging—through frequency-dimension scale translation to apply the novel concept of differential imaging.

The *XY* planar measurement geometry is used for illuminating the metallic vehicle using the ultrawide-band (UWB) imaging radar system [3]. This illumination provides optimization of the induced currents on the vehicular surface, although the vertical monopole antenna is installed perpendicular but connected to the vehicle surface. One of the reasons for using a monopole antenna oriented in the direction of the *Tx*/*Rx* antennas is because, even when the excitation of the monopole mode may be reduced, its mutual coupling with the *Tx*/*Rx* antennas is also reduced and so the multiple reflections between them may certainly be neglected and, at the same time, the surface currents on top of the vehicle due to the antenna should be visible—especially when the *Tx*/*Rx* antennas move out of the perpendicular direction.

**Figure 2:**
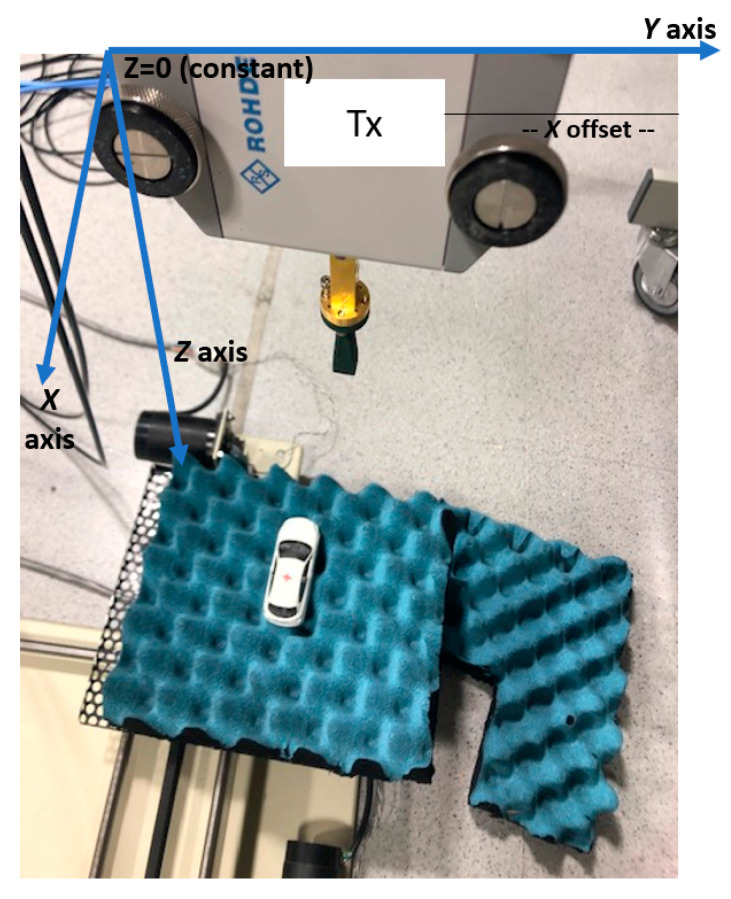
Measurement setup.

The object is placed on a radiofrequency-absorbing material located 0.5 m from the *Tx*/*Rx* antennas. The separation between the *Tx* antenna with a frequency converter and the *Rx* antenna with a frequency converter is 0.1 m, installed in a fixed position in order to avoid any perturbation in the measurement associated with coaxial cables and converter movements. The object under measurement is placed on a positioning table that is capable of moving in two axes (*x*-axis and *y*-axis) with steps of 1 mm [16]. Figure 2 displays the frequency converter with the horn antenna mounted and the positioning table with the metallic car.

The S21 parameter measurements were carried out by sweeping the frequency band from 220 GHz to 330 GHz. The two-frequency converter equipment was manufactured by Rohde & Schwarz (Munich, Germany)^®^, with the model being the ZVA-Z325 Frequency Converter, J-Band WR-03 [17].

Table 1 includes the VNA configuration parameters.

The *Tx* and *Rx* antennas were horns produced by Flann Microwave LTD (Cornwall, United Kingdom) [18]. The selected horn model was the 32240-25, with a bandwidth (BW) from 217 GHz to 330 GHz. The *E* and *H* plane gain was 23.70 dBi at 217 GHz and 26.99 dBi at 330 GHz. The E and H plane beamwidth was 11.9° at 217 GHz and 7.8° at 330 GHz.

**Figure 3:**
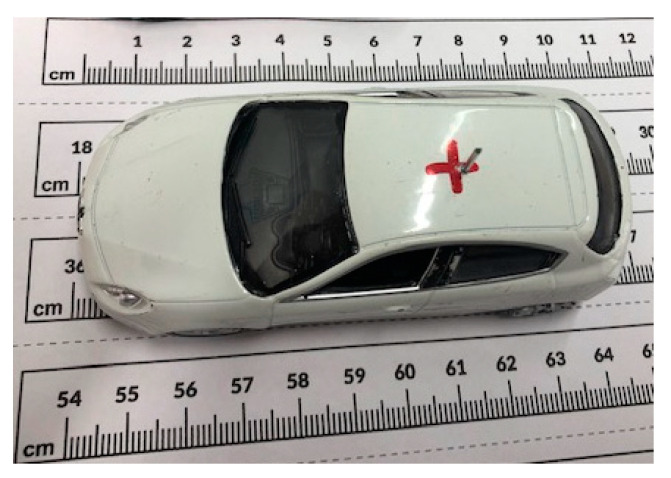
A metallic car with an antenna for differential imaging.

The selected object is a metallic car with a 1:43 scale and 10 cm length. This is a good model for a real car, in terms of both shape and materials. The scale of the car corresponds with the frequency range between 2.5 and 3.8 GHz. A short 15 mm metal wire was placed on top of the car to act as an antenna, as shown in Figure 3. The measurement was performed with (ON state) and without the antenna (OFF state) mounted in the metallic car with the aim of estimating the induced current on the car after using the imaging system to illuminate.

## 3. Results

**Figure 4:**
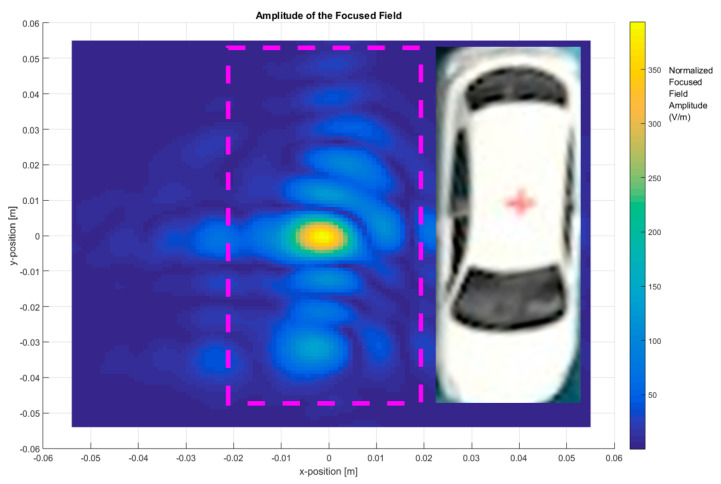
Focused field amplitude of the metallic car without an antenna. Frequency range: 295 GHz–305 GHz (bandwidth (BW) = 10 GHz).

**Figure 5:**
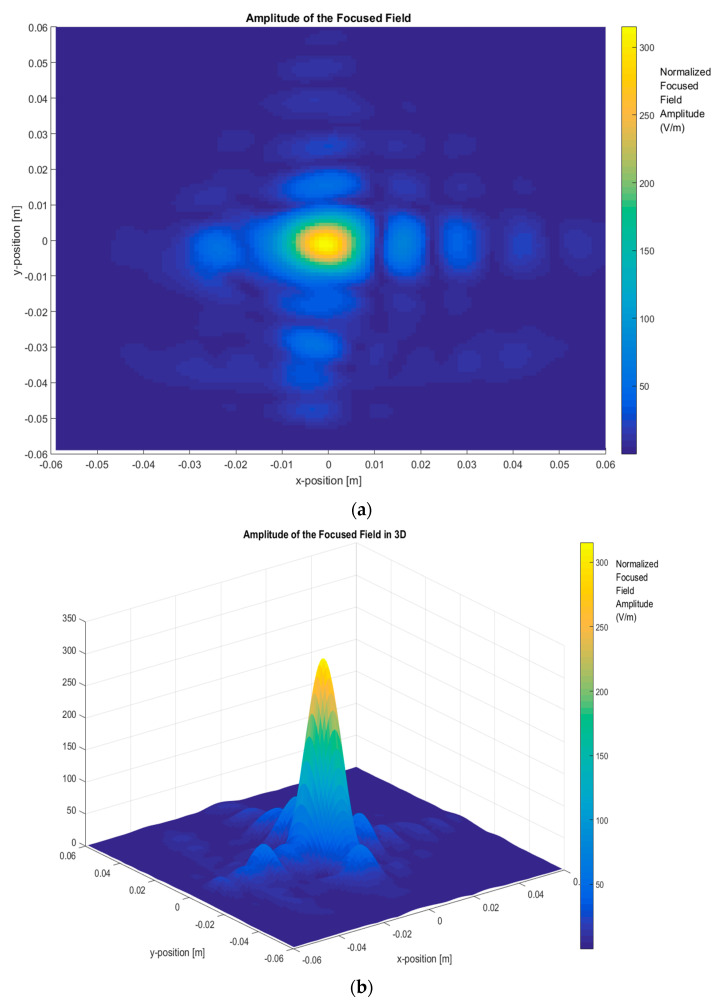
The focused field amplitude of a metallic car without an antenna. Frequency range: 220 GHz–330 GHz (BW = 110 GHz): (**a**) *XY* plane representation; (**b**) 3D representation of the focused field.

**Figure 6:**
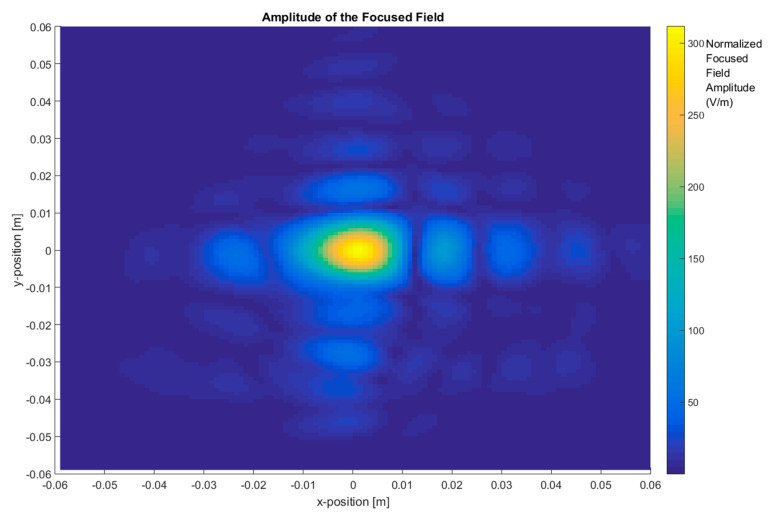
The focused field amplitude of a metallic car with an antenna. Frequency range: 220 GHz–330 GHz (BW = 110 GHz).

**Figure 7:**
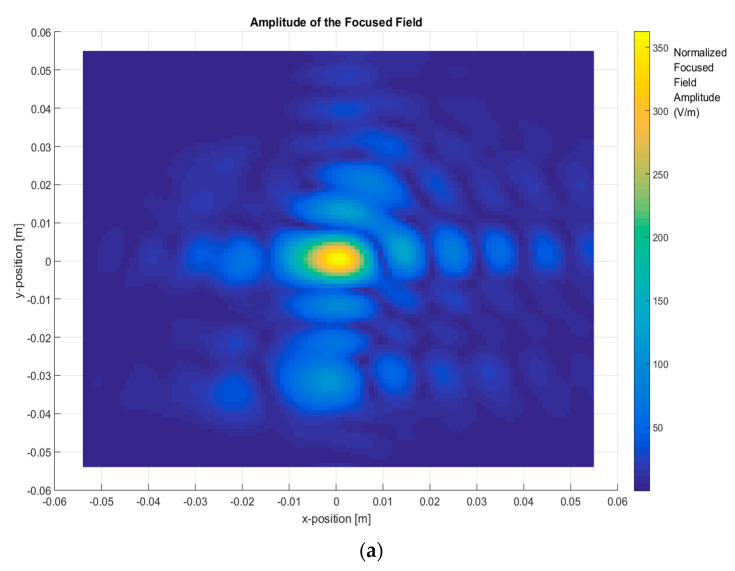

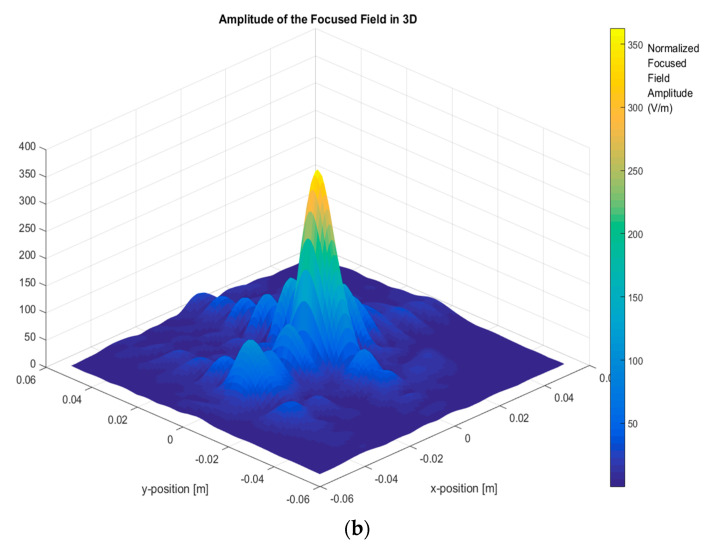
The focused field amplitude of a metallic car with an antenna. Frequency range: 295 GHz–305 GHz (BW = 10 GHz). (**a**) *XY* plane representation; (**b**) 3D representation of the focused field.

In the first step, the total images were obtained based on the two-step process by measuring the scattered fields first and then obtaining the focused field by application of Equation (4) for different frequency ranges and focusing the car with (ON state, short-circuited monopole antenna) and without the antenna (OFF state, open-circuited monopole antenna), as depicted in Figure 4, Figure 5, Figure 6 and Figure 7.

The bandwidth used to reconstruct the image is an important imaging parameter. The first image is shown in Figure 4, which depicts the total image reconstruction without an antenna on top of the metallic car with a frequency of 295 GHz–305 GHz. The top of the metallic car has a curvature that generates a specific focused field distribution.

Then, Figure 5 presents the total image reconstructed without an antenna using a bandwidth of 110 GHz, sweeping from 220 GHz to 330 GHz. This bandwidth provides better resolution performances, meaning the top of the metallic car can be identified in the image as a very clear reconstruction. Additionally, Figure 5b highlights the focused field amplitude value and the distribution due to the reflection in the top of the car, including some minor contributions around the top of the car.

Figure 6 shows the total image reconstructed with the antenna installed on the top and using a bandwidth of 110 GHz, from 220 GHz to 330 GHz. The maximum of the focused fields is identified around the center of the top of the metallic car, where the antenna is installed. The antenna provides reflectivity, but it is masked with the reflectivity of the metallic car top in the graph.

Finally, Figure 7 depicts the total image with the antenna installed on the top, using a bandwidth of 10 GHz around 300 GHz. The shape of the focused field is like that in Figure 4 due to the antenna contribution being added to the reflectivity of the metallic car top.

## 4. Discussion

The measured fields are focused on different image planes along the *z*-axis (*z* = 0.4787 m) where the capability of the system for focusing on different longitudinal distances (along the *z*-axis) and different frequency ranges and bandwidths, over different transversal planes (*XY* planes), may be observed. The experimental results show that using a wide bandwidth (110 GHz) is not required in order to obtain a good performance. The performance was of sufficient quality using 10 GHz bandwidth around 300 GHz. The resolutions would be:

(9) Δz~1ΔB

(10) Δx,Δy~λ0(2sinθ0)

(11) tanθ0~X0Z0 or Y0Z0 

where ΔB is the frequency bandwidth of the measurement, $\lambda_{0}$ is the central frequency wavelength, $\theta_{0}$ is the target angle visualization seen from the measurement distance, and $Z_{0}$ and $X_{0}$, are the scanning distances along the *x*-axis and *y*-axis, respectively.

Differential imaging is obtained by means of calculating the difference of the experimental data (total image obtained from focused field amplitude) measured using the car, including with a monopole antenna short-circuited (ON state) and without the antenna (OFF state or open-circuited antenna). As stated in Section 2, the short-circuited antenna represents the antenna placed on the top of the car, which is in contact with the car’s metal surface.

**Figure 8:**
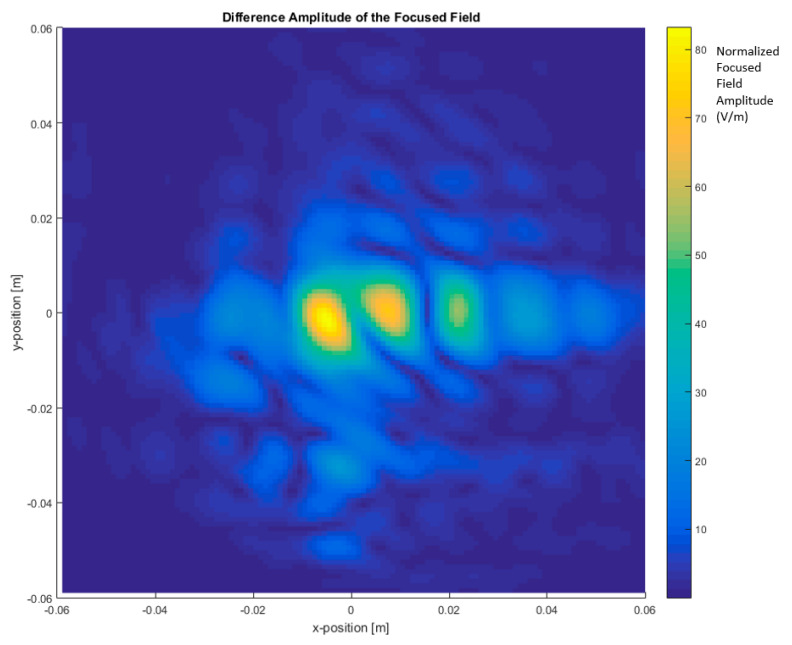
Differential focused field amplitude between the metallic car with and without an antenna. Frequency range: 220 GHz to 330 GHz (BW = 110 GHz).

**Figure 9:**
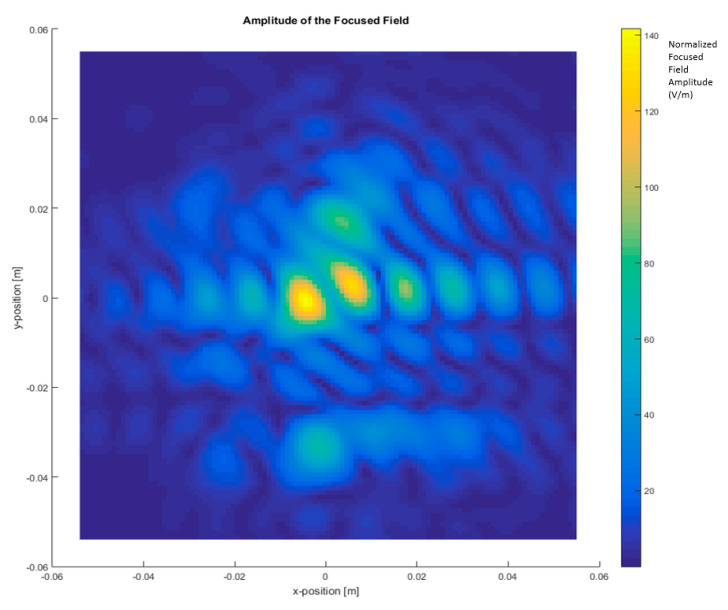
Differential focused field amplitude between the metallic car with and without an antenna. Frequency range: 295 GHz to 305 GHz (BW = 10 GHz).

The objective is to obtain the currents created by the antenna over the surface of the vehicle without getting dazzled (“flash-out”) by the higher intensity of the currents on the antenna itself. A differential image may be produced due to the difference in module between the focused field amplitude images with the antenna (see Figure 6 and Figure 7) and without the antenna (see Figure 4 and Figure 5), resulting in the current distribution of the antenna that is shown in Figure 8 and Figure 9.

In Figure 8, it can be identified that the strong central spot corresponding to the antenna has disappeared and, instead, the nearby currents to the antenna are visualized. The maximum can be clearly identified.

Figure 9 depicts the current distribution calculated as a differential focused field amplitude using a frequency range from 295 GHz to 305 GHz (BW = 10 GHz) to assess influence of bandwidth in differential imaging.

The differential focused field amplitude is higher with a bandwidth of 10 GHz around 300 GHz compared with a BW of 110 GHz from 220 GHz to 330 GHz. This effect is due to the gain variation of the horns across the frequency range. The imaging system performs an averaging through all the bandwidths, and the horn antenna gain variation generates this change in the amplitude, with higher amplitude when the BW is lower. However, the image resolution is better when the BW is greater [3]. If the application requires high resolution, then the BW parameter should be maximized.

## 5. Conclusions

In this work, an application of the approach in the near-field exploration of metallic objects explained in [3] is used to perform the focusing of a metallic car with and without a monopole antenna in order to estimate the induced currents on the vehicle surface using a differential methodology. This method makes it possible to estimate the distribution of the currents induced by the antenna on the vehicle’s surface using a very simple method, which is also combined with the frequency-dimension scale translation.

The technique presented in [3] has been extended to a different measurement scenario in a 3D scan in the terahertz band and by using the differential current concept as both a novel form and a major contribution.

The findings verify that, while distance discrimination increased with the bandwidth, the transverse resolution (*XY* plane) did not change significantly.

The results show promising performance from this technique using differential currents, as depicted in the 300 GHz band. Gaining knowledge of these vehicle-antenna-created currents may offer hints regarding the impact produced by the shapes (borders and corners), discontinuities (slots and windows), or materials (composition or roughness) introduced into the vehicular platform.

## Figures and Tables

**Table 1:** Vector network analyzer (VNA) configuration parameters.

Parameter	Value
Start frequency	220 GHz
End frequency	330 GHz
Sampling points	8192
Intermediate frequency (IF) bandwidth	1 kHz
Emitting power	0 dBm

## References

[1] Chattopadhyay X.G. (2011). Technology, capabilities, and performance of low power terahertz sources. IEEE Trans. Terahertz Sci. Technol..

[2] Siegel P. (2002). Terahertz technology. IEEE Trans. Microw. Theory Technol..

[3] Solano-Perez J.A., Martínez-Inglés M.-T., Molina-Garcia-Pardo J.-M., Romeu J., Jofre L., Rodríguez J.-V., Mateo-Aroca A. (2020). Linear and Circular UWB Millimeter-Wave and Terahertz Monostatic Near-Field Synthetic Aperture Imaging. Sensors.

[4] Friederich F., May K.H., Baccouche B., Matheis C., Bauer M., Jonuscheit J., Moor M., Denman D., Bramble J., Savage N. (2018). Terahertz Radome Inspection. Photonics.

[5] Hao J., Li J., Pi Y. (2018). Three-Dimensional Imaging of Terahertz Circular SAR with Sparse Linear Array. Sensors.

[6] Dobroiu A., Wakasugi R., Shirakawa Y., Suzuki S., Asada M. (2018). Absolute and Precise Terahertz-Wave Radar Based on an Amplitude-Modulated Resonant-Tunneling-Diode Oscillator. Photonics.

[7] Fromenteze T., Decroze C., Abid S., Yurduseven O. (2018). Sparsity-Driven Reconstruction Technique for Microwave/Millimeter-Wave Computational Imaging. Sensors.

[8] King D.D. (1949). Measurement and interpretation of antenna scattering. IEEE Proc. IRE.

[9] Lin D.B., Chu T.H. (1993). Bistatic frequency-swept microwave imaging: Principle, methodology, and experimental results. IEEE Trans. Microw. Theory Technol..

[10] Jofre L., Broquetas A., Romeu J. (2009). UWB Tomographic Radar Imaging of Penetrable and Impenetrable Objects. Proc. IEEE.

[11] Alvarez Y., Rodriguez-Vaqueiro Y., Gonzalez-Valdes B., Mantzavinos S., Rappaport C.M., Las-Heras F., Martínez-Lorenzo J.Á. (2014). Fourier-based Imaging for Multi-static Radar System. IEEE Trans. Microw. Theory Technol..

[12] Kim Y.J., Jofre L., de Flaviis F., Feng M.Q. (2003). Microwave Reflection Tomographic Array for Damage Detection of Civi Structures. IEEE Trans. Antennas Propag..

[13] Broquetas A., Palau J., Jofre L., Cardama A. (1998). Spherical Wave Near-Field Imaging and Radar Cross-Section Measurement. IEEE Trans. Antennas Propag..

[14] Bouazza B. (2020). Millimeter-Wave MIMO Close Range Imaging. Master’s Thesis.

[15] Capdevila S., Jofre L., Romeu J., Bolomey J.C. (2013). Multi-Loaded Modulated Scatter Technique for Sensing Applications. IEEE Trans. Instrum. Meas..

[16] Arrick Robotics—Stepper Motor, Positioning, Automation, Mobile Robots, Resources. http://www.arrickrobotics.com/.

[17] Rohde & Schwarz GmbH & Co. KG. https://www.rohde-schwarz.com.

[18] Homepage Flann—Flann Microwave. http://flann.com/.

